# A Candidate Prognostic Biomarker Complement Factor I Promotes Malignant Progression in Glioma

**DOI:** 10.3389/fcell.2020.615970

**Published:** 2021-02-04

**Authors:** Xiaomin Cai, Wenjin Qiu, Mengshu Qian, Shuang Feng, Chenghao Peng, Jiale Zhang, Yi Wang, Yuhai Wang

**Affiliations:** ^1^Department of Neurosurgery, The 904th Hospital of Joint Logistic Support Force of People's Liberation Army (PLA), Clinical Medical College of Anhui Medical University, Wuxi, China; ^2^Department of Neurosurgery, The Affiliated Hospital of Guizhou Medical University, Guiyang, China; ^3^Department of Emergency, The 904th Hospital of Joint Logistic Support Force of People's Liberation Army (PLA), Clinical Medical College of Anhui Medical University, Wuxi, China; ^4^Department of Encephalopathy, The Third Affiliated Hospital of Nanjing University of Chinese Medicine, Nanjing, China; ^5^Department of Neurosurgery, Tongji Hospital, Tongji Medical College, Huazhong, University of Science and Technology, Wuhan, China; ^6^Department of Neurosurgery, Xijing Hospital, The Fourth Military Medical University, Xi'an, China; ^7^Department of Urology, Affiliated Hospital of Nantong University, Nantong, China

**Keywords:** Glioma, CFI, prognosis, biomarker, invasion, proliferation

## Abstract

**Objectives:** Glioma is the most common and aggressive type of primary central nervous system (CNS) tumor in adults and is associated with substantial mortality rates. The aim of our study was to evaluate the prognostic significance and function of the complement factor I (CFI) in glioma.

**Materials and Methods:** The expression levels of CFI in glioma tissues and the survival of the CFI^high^ and CFI^low^ patient groups were analyzed using The Cancer Genome Atlas (TCGA) database and Genotype-Tissue Expression (GTEx). The correlation between CFI expression and clinicopathological features of glioma was determined by univariate and multivariate Cox regression analyses in the Chinese Glioma Genome Atlas (CGGA) database. The functional role of CFI in glioma was established through routine *in vitro* and *in vivo* assays.

**Results:** CFI is overexpressed in glioma and its high levels correlated with poor outcomes in both TCGA and CGGA datasets. Furthermore, CFI was identified as an independent prognostic factor of glioma in the CGGA database. CFI knockdown in glioma cell lines inhibited growth *in vitro* and *in vivo*, whereas its ectopic expression increased glioma cell proliferation, migration, and invasion *in vitro*. CFI protein levels were also significantly higher in the glioma tissues resected from patients and correlated to worse prognosis.

**Conclusions:** CFI is a potential prognostic biomarker in glioma and drives malignant progression.

## Introduction

Gliomas are the most common and aggressive type of primary central nervous system (CNS) tumor in adults (Furnari et al., 2007; Ostrom et al., 2014; Lapointe et al., 2018). Despite improvements in surgical resection, radio-chemotherapy, immunotherapy, and molecular targeted therapies, the 5-year survival rates of glioma remain dismal (Omuro and DeAngelis, 2013; Keunen et al., 2014; Reifenberger et al., 2017; Kieran et al., 2019). Recent studies showed that the combination of molecular features and histological parameters has superior diagnostic and prognostic accuracy compared to histological classification alone (Nutt et al., 2003; Shirahata et al., 2007; Hoshide and Jandial, 2016; Jiang et al., 2020). For instance, expression of 1p/19q genes is a potential prognostic marker in 1p/19q non-codeletion gliomas, whereas isocitrate dehydrogenase (IDH) levels have prognostic value in wild-type IDH gliomas (Calvert et al., 2017; Chai et al., 2019a). Given the molecular heterogeneity of gliomas, it is necessary to identify novel prognostic biomarkers and therapeutic targets (Hoshide and Jandial, 2016; Louis et al., 2016).

The complement system plays a crucial role in the immune response against pathogens by augmenting the ability of antibodies and phagocytes to clear microbes and damaged cells (Merle et al., 2015; Afshar-Kharghan, 2017). It consists of plasma proteins, secreted from the liver, as well as cell membrane-bound proteins that opsonize pathogens and induce a series of inflammatory responses (Kolev et al., 2014; Yu et al., 2014; Morgan et al., 2016). The complement system can be activated by the classical, lectin, and alternative pathways (Ricklin et al., 2010), all of which culminate in the enzymatic cleavage of C3 and C5 into active fragments that form the membrane attack complex (MAC) and eventually trigger cell lysis (Gros et al., 2008; Strainic et al., 2008; Roumenina et al., 2019). A myriad of soluble membrane-bound inhibitory molecules control the complement cascade and minimize the destructive effects of aberrant complement activation.

The complement factor I (CFI) is a serine protease that inactivates the complement cascade by degrading C4b and C3b (Nilsson et al., 2010) in the presence of cofactors like C4b-binding protein (C4BP) (Gigli et al., 1979), complement factor H (CFH) (Weiler et al., 1976), membrane cofactor protein (MCP) (Seya et al., 1986), and complement receptor 1 (CR1) (Medof and Nussenzweig, 1984). The functional CFI protein comprises of a 50-kDa heavy chain and a 38-kDa light chain that are linked covalently by a disulfide bond (Sanchez-Gallego et al., 2012). Recent studies have correlated CFI expression with cancer prognosis. Riihila et al. found that overexpression of CFI in cutaneous squamous cell carcinoma was associated with increased tumor malignancy and progression (Riihila et al., 2015). Likewise, Okroj et al. showed that high levels of CFI in breast cancer portended poor prognosis (Okroj et al., 2015). However, the role of CFI in gliomas is largely unknown.

In the present study, we identified CFI as an independent prognostic factor of gliomas using integrated bioinformatics analyses. CFI was highly expressed in glioma cell lines and tissues, and its knockdown significantly inhibited glioma cell proliferation, migration, and invasion *in vitro* and *in vivo*. In addition, glioma patients with high expression of CFI had worse prognosis compared to patients expressing low CFI. Taken together, CFI serves as an independent prognostic biomarker in glioma and contributes to tumor malignant progression.

## Materials and Methods

### RNA-seq Data and Bioinformatics Analysis

RNA-seq data of CFI in multiple tumor tissues and corresponding normal tissues were obtained from The Cancer Genome Atlas (TCGA; http://cancergenome.nih.gov/) and Genotype-Tissue Expression (GTEx; https://www.gtexportal.org/home/datasets) and analyzed using R programming language. Gene Expression Profiling Interactive Analysis (GEPIA; http://gepia.cancer-pku.cn/index.html) was used to analyze survival differences between the CFI^high^ and CFI^low^ glioblastoma multiform (GBM) and low-grade glioma (LGG) patients. The glioma datasets with mRNA expression and clinical data, including PRS_type, WHO grade, age, chemotherapy status, IDH_mutation_status, 1p/19q_codeletion_status, and histology grade, were acquired from the Chinese Glioma Genome Atlas (CGGA; http://www.cgga.org.cn/) (Dataset ID: mRNAseq_693 and mRNAseq_325). The correlation between CFI expression and clinical features was evaluated by Cox regression analyses. Variables with *P* < 0.05 in the univariate analysis were further confirmed in the multivariate analysis.

### Gene Set Enrichment Analysis

The gene set enrichment analysis (GSEA) tool (Han et al., 2019) was used to identify enriched gene sets according to their association with CFI expression level. Gene set permutations were performed 1,000 times to screen for the CFI-related significant biological pathways. A normalized *P*-value <0.05 and FDR *q*-value <0.05 were considered statistically significant.

### Evaluation of Independent Prognostic Indicator

The prognostic value of each clinical parameter listed above was determined by the receiver operating characteristic (ROC) analysis using the R “survivalROC” package. The sensitivity and specificity of prognostic signatures in predicting the overall survival (OS) were evaluated in terms of the area under the curve (AUC) of ROC. A prognostic nomogram was then constructed using the rms package of R in order to predict 1-, 2-, and 3-year OS of glioma patients based on the CGGA dataset. Pearson's correlation analysis was performed to identify CFI-related genes using the CGGA dataset. *P* < 0.05 was considered as statistically significant.

### Cell Lines and Human Tissue Samples

The normal human astrocyte cell line NHA was purchased from Lonza, and human glioma cell lines A172, U118, LN229, U251, T98G, and U87 were obtained from the American Type Culture Collection (ATCC). All cell lines were cultured in as previously described (Cai et al., 2020). A total of 134 GBM tissues, 30 glioma tissues (grades I–III), and 9 paired normal brain tissues were collected from the First Affiliated Hospitals of Nanjing Medical University (Nanjing, China) and 904th Hospital of Joint Logistic Support Force of People's Liberation Army (Wuxi, China), from 2014 to 2019. The tissues were snap frozen in liquid nitrogen after storing at −80°C overnight. The clinical and pathological characteristics for 134 GBM patients are shown in Supplementary Table 2. The tissue microarray (TMA) for immunohistochemical staining was constructed based on 9 NBTs, 7 grade I, 8 grade II, 15 grade III, and 18 grade IV glioma specimens. The glioma tissues were confirmed histologically by two independent neuropathologists according to the WHO criteria. The study protocol was reviewed and approved by The Institutional Review Board of Nanjing Medical University and Anhui Medical University. All patients had signed written informed consent before the surgery.

### Quantitative Real-Time PCR and Western Blotting

Quantitative real-time PCR (qRT-PCR) and western blotting were performed as described previously (Cai et al., 2020). The primers for CFI were as follows: forward: 5′-CTCAGCAGAGACAAAGAC-3′, reverse: 5′-GTGGAAGCACAGAAATAAC-3′. GAPDH served as the internal control and the primers were as follows: forward 5′-GAAGGTGAAGGTCGGAGTC-3′, reverse: 5′-GAAGATGGTGATGGGATTTC-3′. Antibody against CFI was obtained from Novus Biologicals. Anti-VEGFR2, anti-AKT, anti-phospho-AKT (Thr308), anti-p38 MAPK, and anti-phospho-p38 (Thr180/Thr182) primary antibodies were obtained from Cell Signaling Technology. Anti-FAK and anti-phospho-FAK (Tyr576/577) primary antibodies were purchased from Abcam. Anti-β-actin antibody, which was purchased from Sigma-Aldrich, acted as the negative control. All experiments were conducted in triplicate and repeated at least three times.

### Immunohistochemistry

Briefly, paraffin-embedded tissue sections (4-μm-thick) were baked at 60°C for 1 h, deparaffinized in xylene, and rehydrated in graded concentrations of ethanol (100, 95, and 85% for 5 min each). The sections were placed in pH 6.0 citric buffer for 20 min, treated with 3% hydrogen peroxide in phosphate-buffered saline, and incubated with goat serum. Each section was incubated with primary and secondary antibodies separately. Chromogen was added and the specimens were counterstained with hematoxylin. Immunohistochemical images were taken using a Leica DM IRE2 microscope (Leica Microsystems Imaging Solutions Ltd., Cambridge, United Kingdom) and analyzed by Image-Pro Plus v6.0 software (Media Cybernetics Inc., Bethesda, MD, USA). The integrated optical density (IOD) was calculated according to the following grading system: staining extensity was categorized as 0 (≤5% positive cells), 1 (>5% and ≤25% positive cells), 2 (>25% and ≤50% positive cells), or 3 (>50% positive cells), and staining intensity was categorized as 0 (negative), 1 (weak), 2 (moderate), or 3 (strong). All the samples were examined and scored by two experienced pathologists. Cases with discrepancies in the scores were discussed to reach a consensus.

### Plasmid Construction and Transfection

The pCDNA3.1-CFI recombinant plasmid was constructed by cloning the CFI cDNA into the pCDNA3.1 vector. Two GBM cell lines stably expressing shCFI or shCtrl were established using a lentiviral packaging kit (Santa Cruz Biotechnology) and selected with 5 mg/ml puromycin 48 h after transfection. The cells were transfected using Lipofectamine 3000 (Invitrogen) according to the manufacturer's protocol.

### CCK-8 Assay

The proliferation rate of glioma cells was examined by the CCK-8 kit (Beyotime) following the manufacturer's instructions. Briefly, the suitably transfected cells were seeded into 96-well plates at the density of 5 × 10^3^ cells/well, and 10 μl CCK-8 reagent was added per well at 24, 48, 72, and 96 h of culture. The absorbance at 450 nm was measured to calculate the percentage of viable cells.

### Colony Formation Assay

The cells were seeded into a six-well plate at the density of 5 × 10^2^ cells/well and cultured for 14 days. The colonies were washed thrice with PBS, fixed with 100% methanol for 20 min, and stained with 1% crystal violet in 20% methanol for 15 min. After washing thrice with PBS, the number of colonies was counted and the averages were calculated.

### Wound Healing Assay

The suitably treated glioma cells were seeded onto a coverslip in six-well plates at the density of 3 × 10^5^ cells/well. The monolayer was scratched using a sterile 20-μl pipette tip, and the wound area was photographed at 0 and 24 h under a light microscope (Leica, Wetzlar, Germany). The migration index was calculated in terms of wound coverage.

### Invasion Assay

The invasion assay was performed as described previously (Cai et al., 2020) using 24-well BD Matrigel Invasion Chambers (BD Biosciences). Briefly, 5 × 10^4^ suitably treated glioma cells were seeded in the upper wells of the chamber in serum-free media. The lower chamber wells contained DMEM supplemented with 20% FBS. After incubation for 24 h, non-invading cells were scraped off and invading cells on the bottom cells were fixed and stained. The number of invading cells was counted in three representative fields per sample.

### Orthotopic Xenograft Studies

An orthotopic glioma model was established by intracranially injecting 5 × 10^6^ U251 cells into 4−6-week-old immunodeficient mice. Tumor growth was monitored by *in vivo* bioluminescence imaging. The tumor length (*L*) and width (*W*) were measured with calipers, and the volume (*V*) was calculated as (*L* × *W*^2^)/2. Mice were sacrificed when they showed signs of ill health such as weight loss, rough coat, etc. Brains were harvested and further fixed with 4% paraformaldehyde at 4°C overnight. All animal studies were approved by the Animal Welfare Ethical Review Committee of Nanjing Medical University and Anhui Medical University and conducted in accordance with the National Guidelines for the Care and Use of Laboratory Animals.

### Statistical Analysis

SPSS 24.0 (IBM, Chicago, USA), R3.3.1 (https://www.r-project.org/), and GraphPad Prism 7.0 programs were used for statistical analyses. The association between clinicopathologic features and CFI was evaluated with the Wilcoxon signed rank test and logistic regression. Pearson's correlation analysis was used to identify CFI-related genes in the CGGA database. Univariate and multivariate Cox regression analyses were used to evaluate the relationship between the clinical variables and OS. The nomogram model was created using the rms package of R software. ROC and AUC were calculated using the package of “survivalROC” in R. All experimental data was expressed in the format of mean ± standard deviation. Student's *t* test was used for pairwise comparison, and one-way analysis of variance and Cox regression analysis were used for multiple group comparison. Survival was analyzed using the Kaplan–Meier method with GraphPad Prism 7. *P* < 0.05 was considered statistically significant.

## Results

### CFI Was Overexpressed in Gliomas and Associated With Poor Outcome

**Figure 1:**
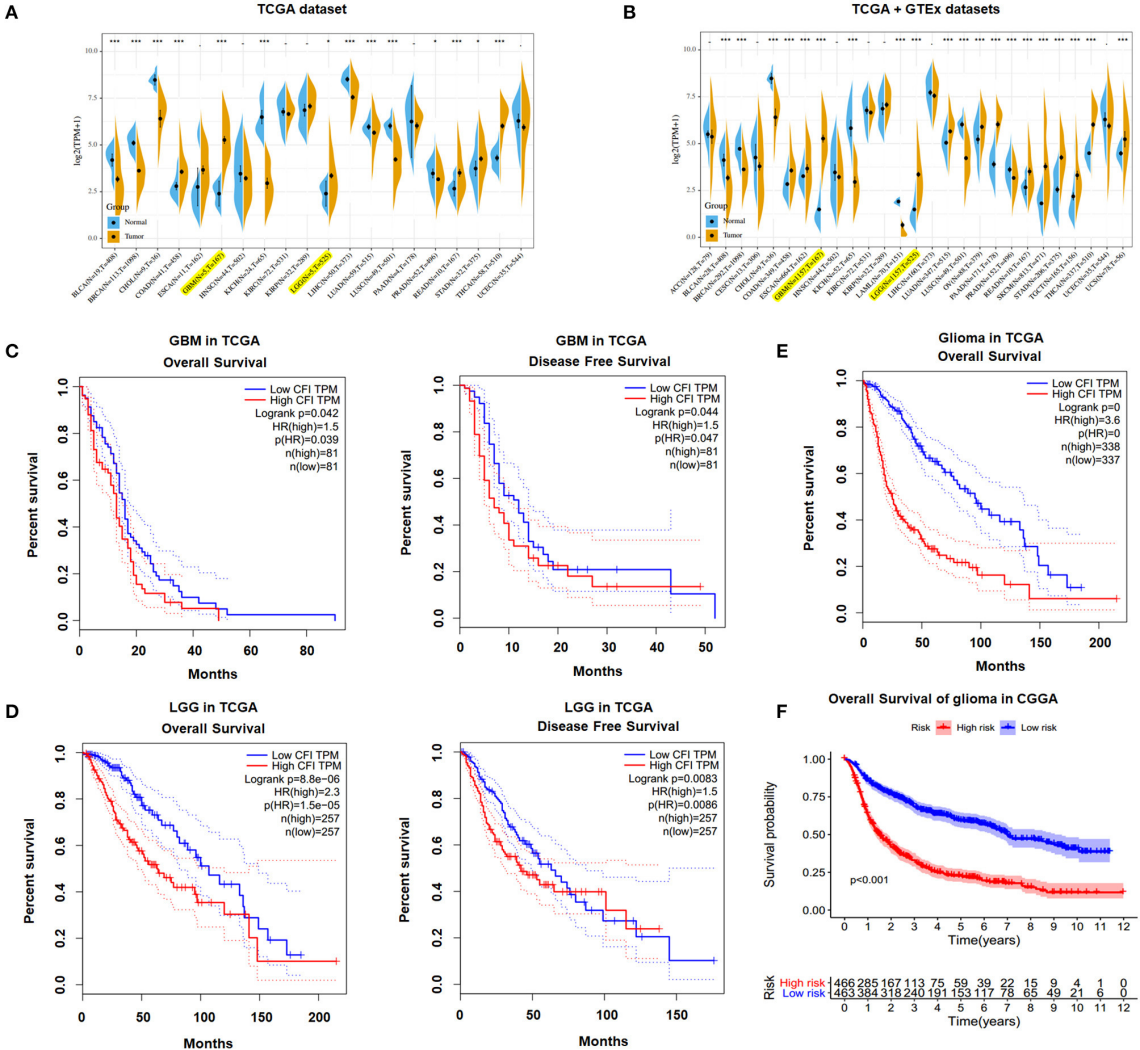
Complement factor I (CFI) was overexpressed in gliomas and associated with poor outcome. **(A)** CFI expression levels in different tumor types and corresponding normal tissues from The Cancer Genome Atlas (TCGA) database. The yellow and blue plots respectively correspond to the tumor and paired normal tissues. **(B)** The expression levels of CFI in different tumor types and corresponding normal tissues based on TCGA and Genotype-Tissue Expression (GTEx) databases. **(C)** Overall survival (OS) (left panel) and disease-free survival (DFS) (right panel) of CFI^high^ and CFI^low^ glioblastoma multiform (GBM) patients in TCGA database. **(D)** OS (left panel) and DFS (right panel) of CFI^high^ and CFI^low^ low-grade glioma (LGG) patients in TCGA database. **(E)** OS of all glioma patients stratified by CFI expression level in TCGA database. **(F)** OS of all glioma patients stratified by CFI expression level in the Chinese Glioma Genome Atlas (CGGA) database.

We identified CFI as a potential biomarker of glioma on the basis of bioinformatics analysis and the differential expression between gliomas and corresponding normal tissue samples (data not shown). We analyzed the CFI expression profile across diverse tumors in TCGA database and found that CFI was significantly overexpressed in COAD (colon adenocarcinoma), GBM, LGG, READ (rectum adenocarcinoma), STAD (stomach adenocarcinoma), and THCA (thyroid carcinoma) relative to the corresponding normal samples (Figure 1A). Analysis of both TCGA and GTEx datasets indicated significantly higher levels of CFI in 10 tumor types compared to the normal specimens (Figure 1B). Furthermore, higher CFI expression was associated with significantly shorter OS and disease-free survival (DFS) in both GBM (Figure 1C) and LGG (Figure 1D) patients of TCGA cohort. We then stratified all glioma patients in TCGA database into the CFI^high^ and CFI^low^ groups and found that CFI overexpression correlated to shorter OS in all gliomas (Figure 1E). Consistent with this, high CFI expression level also predicted worse prognosis for all glioma patients in the CGGA database (Figure 1F). Taken together, CFI was overexpressed in gliomas and associated with poor prognosis.

Furthermore, we tried to investigate the expression and prognostic value of CFI in glioma subgroups in the CGGA dataset according to WHO 2016 classification (Louis et al., 2016; Jiang et al., 2020). As shown in Supplementary Figures 1A–C, the CFI expression was significantly elevated in IDH wildtype grade II glioma, IDH mutant and 1p/19q non-codeleted grade III glioma, and IDH wildtype GBM compared to corresponding control groups. Then, we evaluated the prognosis stratification ability of the CFI in all six glioma subgroups. We divided patients into high-risk and low-risk groups in various groups of gliomas by their respective CFI expression. We found that patients in high-risk groups had shorter OS than low-risk groups in IDH mutant grade II glioma and IDH mutant GBM subgroups (Supplementary Figures 1D,H). But, we observed no statistically significant difference in patients' OS between high-risk and low-risk groups in IDH wildtype grade II glioma, IDH mutant and 1p/19q codeleted or non-codeleted grade III glioma, and IDH wildtype GBM (Supplementary Figures 1E–G,I). The limited number of cases may lead to insignificant *p*-value.

### CFI Expression Is Correlated to Clinical Characteristics of Glioma

**Figure 2:**
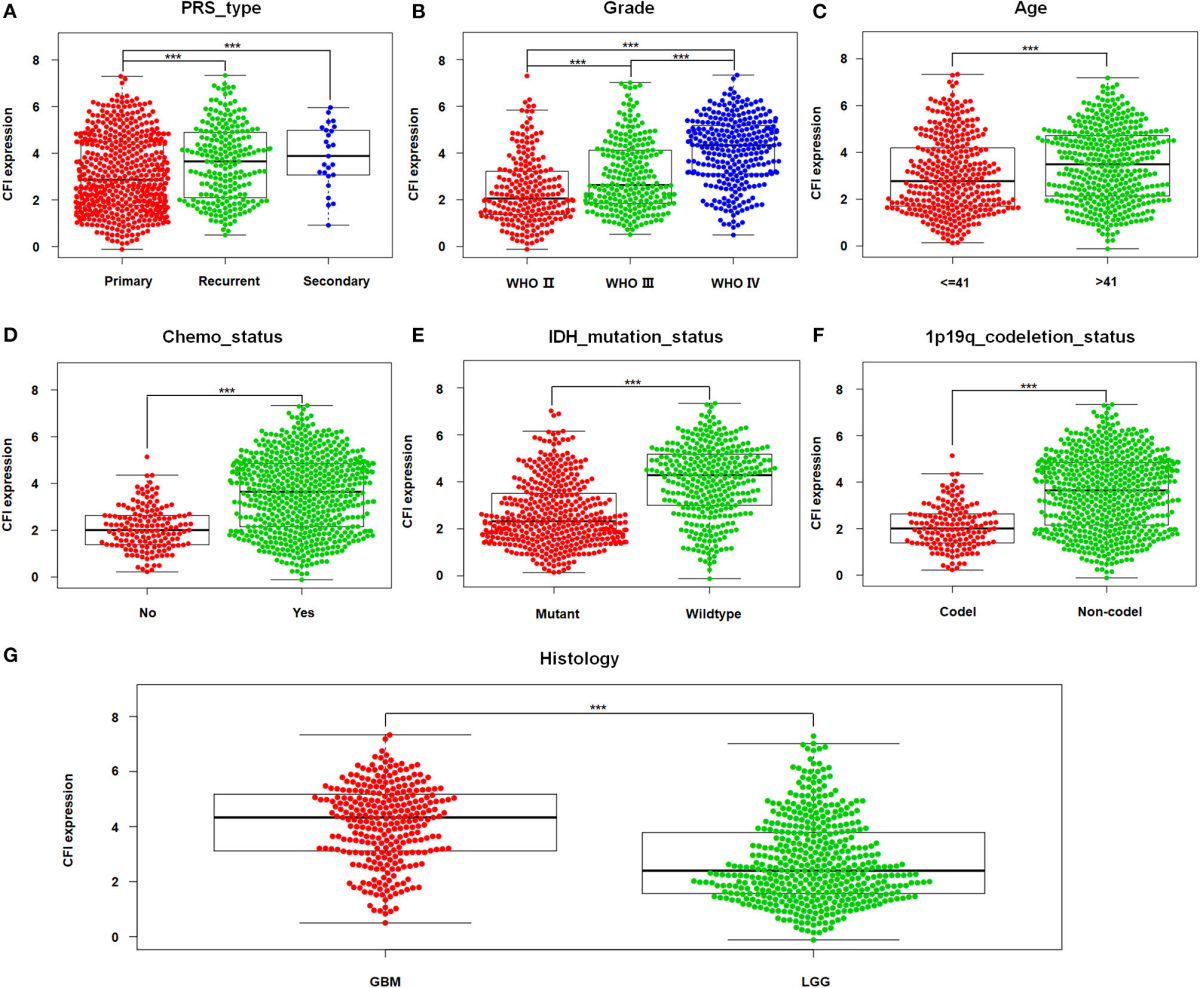
CFI expression is associated with clinicopathological features. **(A–G)** The correlation between CFI expression and clinical variables [primary/recurrent/secondary (PRS) type, WHO grade, age, chemotherapy status, isocitrate dehydrogenase (IDH) mutation status, 1p/19q co-deletion status, and histological grade] in the CGGA dataset. ***P < 0.001.

The correlation between CFI expression in gliomas and the clinicopathological features were next analyzed in the CGGA dataset. As shown in Figure 2A, patients with recurrent or secondary gliomas expressed higher levels of CFI compared to those with primary gliomas. In addition, CFI expression was positively correlated with the WHO tumor grade (Figure 2B), age (≥41 years), and chemotherapy status (Figures 2C,D). Consistent with this, CFI expression was significantly higher in the GBM patients compared to LGG patients (Figure 2G). Furthermore, glioma samples with IDH mutation or 1p/19q co-deletion expressed lower levels of CFI compared to the corresponding non-mutated samples (Figures 2E,F). Collectively, CFI expression is strongly associated with the clinicopathological features of glioma, including PRS type (primary/recurrent/secondary), WHO grade, age, chemotherapy status, IDH mutation status, 1p/19q co-deletion status, and histology grade, and is therefore a promising biomarker for prognosis as well as a therapeutic target.

### CFI Is an Independent Prognostic Indicator of OS in Gliomas

**Figure 3:**
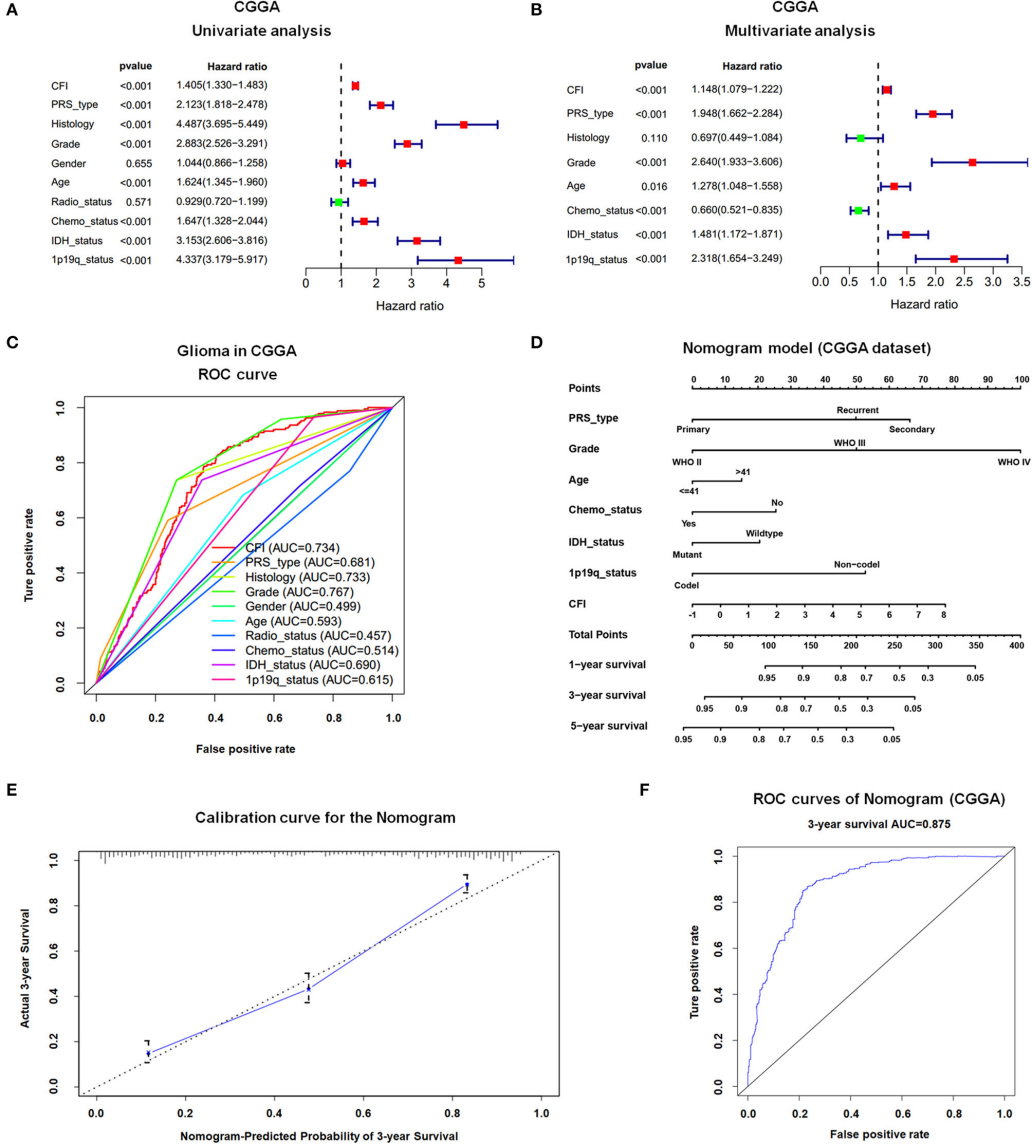
Prognostic value of CFI expression in gliomas. **(A,B)** Forest plot displaying results of the univariate **(A)** and multivariate **(B)** Cox regression analysis of CFI expression and clinical parameters in predicting the OS of glioma patients in the CGGA dataset. **(C)** The receiver operating characteristic (ROC) curves of 10 clinical variables for predicting OS of glioma patients in CGGA dataset. **(D)** Prognostic nomogram combining CFI expression and six clinical variables to predict 1-, 3-, and 5-year OS using CGGA dataset. **(E)** Calibration curve of the nomogram for predicting 3-year OS in the CCGA dataset. **(F)** ROC curves showing the predictive accuracy of the nomogram model for 3-year OS in CGGA dataset.

In a previous study, we found that glioma patients expressing high levels of CFI had significantly worse OS and DFS compared to the CFI^low^ patients in TCGA and CGGA databases. In this study, we performed univariate and multivariate Cox regression analyses on the CGGA dataset to determine the prognostic value of CFI expression and other clinical variables in gliomas (Table 1). Univariate Cox regression analysis indicated that CFI expression, PRS type, histology, WHO grade, age, chemotherapy status, IDH mutation, and 1p19q co-deletion were significantly correlated with the OS of glioma patients, whereas gender and radiotherapy status did not show any correlation (Figure 3A). Seven parameters were further identified as independent prognostic factors of OS by multivariate analysis, including CFI expression [*P* < 0.001, hazard ratio (HR) = 1.149, 95% confidence interval (CI) = 1.079–1.222], PRS type (*P* < 0.001, HR = 1.930, 95% CI = 1.643–2.268), WHO grade (*P* < 0.001, HR = 2.676, 95% CI = 1.959–3.655), age (*P* = 0.013, HR = 1.290, 95% CI = 1.056–1.576), chemotherapy status (*P* = 0.001, HR = 0.672, 95% CI = 0.529–0.855), IDH mutation (*P* = 0.002, HR = 1.467, 95% CI = 1.156–1.861), and 1p19q co-deletion (*P* < 0.001, HR = 2.299, 95% CI = 1.640–3.223) (Figure 3B).

**Table 1:** Cox regression analysis of CFI expression level as a prognostic indicator of gliomas using the Chinese Glioma Genome Atlas Network (CGGA) database.

**Variable**		**Univariate analysis**			**Multivariate analysis**	
	**HR**	**95% CI**	***P***	**HR**	**95% CI**	***P***
**Overall survival**						
CFI	1.405	1.330–1.483	0.000	1.148	1.079–1.222	0.000
PRS_type	2.123	1.818–2.478	0.000	1.948	1.662–2.284	0.000
Histology	4.487	3.695–5.449	0.000	0.697	0.449–1.084	0.110
Grade	2.883	2.526–3.291	0.000	2.640	1.933–3.606	0.000
Gender	1.044	0.866–1.258	0.655			
Age (years)	1.624	1.345–1.960	0.000	1.278	1.048–1.558	0.016
Radio_status	0.929	0.720–1.199	0.571			
Chemo_status	1.647	1.328–2.044	0.000	0.660	0.521–0.835	0.001
IDH_status	3.153	2.606–3.816	0.000	1.481	1.172–1.871	0.001
1p19q_status	4.337	3.179–5.917	0.000	2.318	1.654–3.249	0.000

*HR, hazard ratio; CI, confidence interval*.

To further elucidate the predictive accuracy of the above parameters for OS in glioma patients, we next conducted an ROC analysis. The AUC of CFI was 0.734 compared to 0.681, 0.733, 0.499, 0.593, 0.457, 0.514, 0.69, and 0.615 calculated for PRS type, histology, gender, age, radiotherapy status, chemotherapy status, IDH mutation status, and 1p19q co-deletion status, respectively. Thus, CFI can predict the OS of glioma patients with moderately high sensitivity and specificity (Figure 3C). However, since CFI expression alone is insufficient to evaluate survival, we constructed a nomogram incorporating the seven independent prognostic factors to quantitatively estimate the 1-, 3-, and 5-year OS possibility of patients with glioma. The survival probability of each patient can be calculated by adding up the scores of each parameter. As shown in Figure 3D, WHO grade and CFI expression contributed the most to prognosis, followed by PRS type, 1p19q co-deletion status, chemotherapy status, IDH status, and age. The total score can be easily calculated by adding up the scores for every parameter to evaluate the survival probability of each patient. The calibration curve for the 3-year OS showed an optimal concordance between the prediction by nomogram and actual observation in CGGA dataset (Figure 3E). However, the calibration curves for the 1- and 5-year OS were not satisfactory due to some limitations of nomogram itself (data not shown). The AUC of the 3-year OS prediction for the nomogram was 0.875 (Figure 3F). The *C*-index of the risk score was 0.769. Thus, the nomogram showed moderate sensitivity and specificity for predicting 3-year OS of glioma patients.

### CFI Expression Is Related to Cancer-Related Signaling Pathways and Genes

To further investigate the potential function of CFI in glioma, we conducted Gene Set Enrichment Analysis (GSEA) using the Kyoto Encyclopedia of Genes and Genomes (KEGG) sets (c2.cp.kegg.v6.2.symbols). The gene sets with normalized *P*-value <0.05 and FDR *q*-value <0.05 were considered significant. As shown in Supplementary Table 1, JAK/STAT, NOD-like receptor, pathways in cancer, T cell receptor, and vascular endothelial growth factor (VEGF) were significantly enriched in the CFI^high^ gliomas (Supplementary Figure 2) and therefore may be involved in mechanisms underlying CFI upregulation in glioma. We next analyzed the correlation between the expression levels of CFI and other genes using the CGGA dataset. Ten genes showed the maximum positive correlation with the expression level of CFI, including ANXA1 (Pearson's *r* = 0.845, *P* < 0.001), ANXA2 (Pearson's *r* = 0.822, *P* < 0.001), C1R (Pearson's *r* = 0.849, *P* < 0.001), C1S (Pearson's *r* = 0.838, *P* < 0.001), CASP4 (Pearson's *r* = 0.805, *P* < 0.001), CP (Pearson's *r* = 0.801, *P* < 0.001), PLAU (Pearson's *r* = 0.805, *P* < 0.001), PROS1 (Pearson's *r* = 0.795, *P* < 0.001), SERPINA3 (Pearson's *r* = 0.802, *P* < 0.001), and TNFRSF12A (Pearson's *r* = 0.795, *P* < 0.001) (Supplementary Figures 3A–J). Furthermore, these 11 genes (CFI, ANXA1, ANXA2, C1R, C1S, CASP4, CP, PLAU, PROS1, SERPINA3, and TNFRSF12A) were also positively correlated with each other (Supplementary Figure 3K). These findings suggest that the co-expressing genes may be involved in CFI-induced tumorigenesis.

Recent studies suggested that VEGF signaling is a complex process involving GBM growth stimulation or inhibition (Kil et al., 2012; Lu et al., 2012; Szabo et al., 2016). Additionally, GBM inevitably progressed during anti-VEGF therapy, such as bevacizumab, through adapting and using alternative signaling pathways to sustain tumor growth (Lu et al., 2012). In this study, we sought to gain insight into the role of CFI in VEGF pathway by immunoblot and the results showed that phosphorylation level of focal adhesion kinase (FAK) at tyrosine sites (Y576 and Y577) was markedly decreased after CFI depletion among various receptor molecules including VEGF receptor 2 (VEGFR2), FAK, protein kinase B (AKT), and p38 mitogen-activated protein kinase (p38 MAPK) (Supplementary Figure 4). Whereas, the total protein level of FAK was not influenced, suggesting that CFI may contribute, at least in part, to FAK activation in whole VEGF signaling pathway.

### CFI Is Overexpressed in Glioma Tissues and Cell Lines and Portends Poor Prognosis

**Figure 4:**
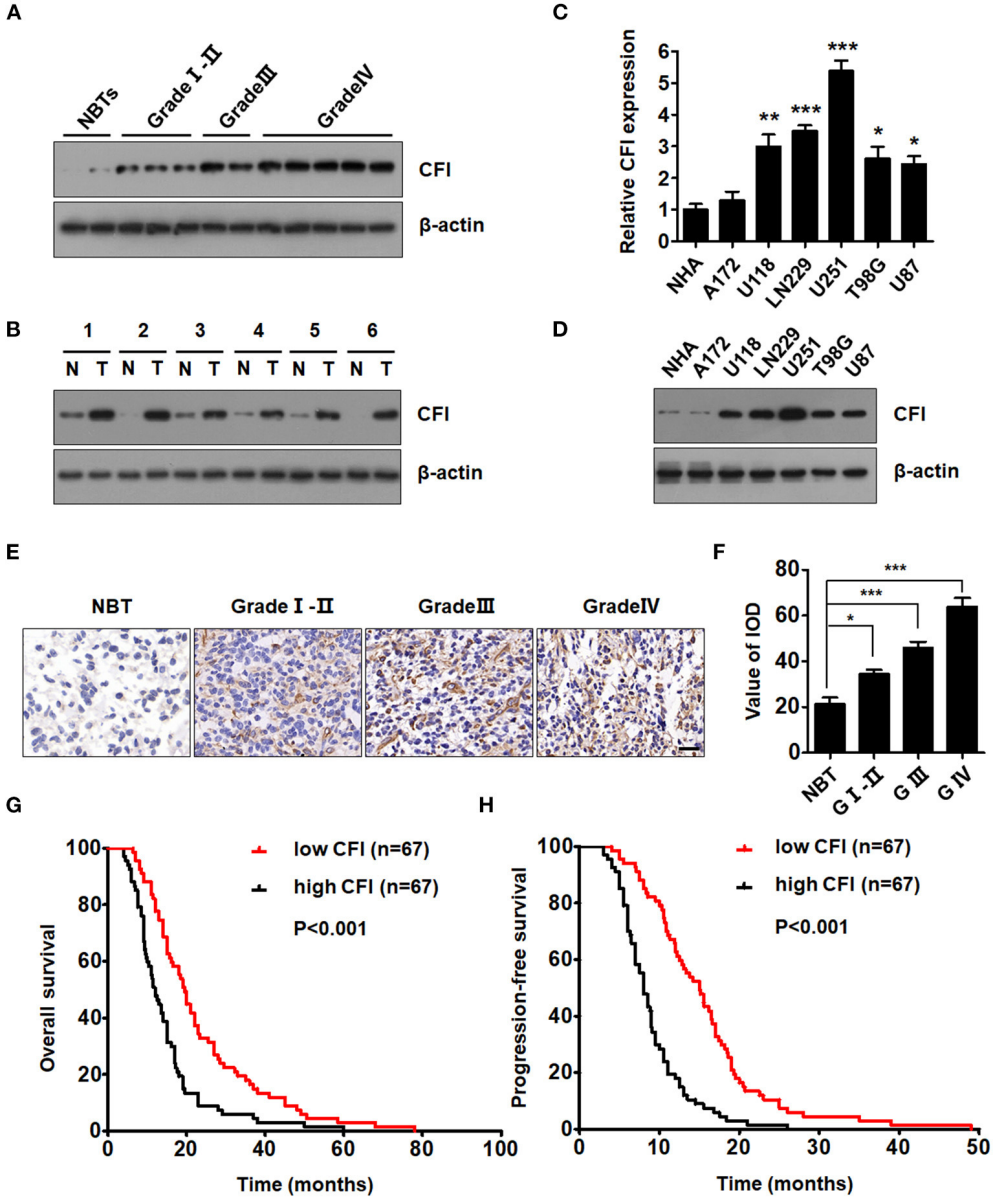
CFI is overexpressed in glioma tissues and cell lines and portends poor prognosis. **(A)** Representative immunoblots showing CFI protein expression in glioma samples of different grades and normal brain tissues (NBTs). β-actin was used as a negative control. **(B)** Immunoblot showing CFI levels in six paired glioma specimens and NBTs. β-actin was used as the loading control. **(C)** CFI mRNA expression level in normal human astrocytes (NHA) and six glioma cell lines (A172, U118, LN229, U251, T98G, and U87). *P < 0.05, **P < 0.01, ***P < 0.001. Transcript levels were normalized to GAPDH expression. **(D)** Immunoblot showing CFI protein level in NHA and glioma cell lines. β-actin was used as the loading control. **(E)** Representative immunohistochemistry (IHC) images showing CFI expression in different grades of glioma samples and NBTs. **(F)** Value of integrated optical density (IOD) showing CFI expression levels in the tissue microarray (NBT 9, Grade I-II 15, Grade III 15, Grade IV 18). *P < 0.05, ***P < 0.001. **(G,H)** The OS **(G)** and progression-free survival (PFS) **(H)** of 134 GBM patients with low (*n* = 67) and high (*n* = 67) CFI expression.

We validated the bioinformatics data on CFI expression level with six paired GBM and NBT specimens, as well as in glioma specimens of different grades. As shown in Figure 4A, CFI protein level increased through glioma grades I–II, III, and IV compared to the NBTs. Furthermore, the expression level of CFI was markedly elevated in the GBM specimens relative to the NBTs (Figure 4B). The expression level of CFI mRNA was also detected in normal human astrocytes (NHAs) and six GBM cell lines (A172, U118, LN229, U251, T98G, and U87), and all GBM cells except A172 expressed markedly higher levels of CFI mRNA compared to the NHAs (Figure 4C). CFI protein expression levels also showed similar trends (Figure 4D). The western blotting results were confirmed by immunohistochemistry, and the glioma tissues showed significantly higher *in situ* expression of CFI compared to the NBTs. Notably, grade IV gliomas had the strongest CFI expression (Figures 4E,F). Consistent with the bioinformatics results, Kaplan–Meier survival analysis suggested that patients with high CFI expression level had shorter overall and progression-free survival compared to those with low CFI expression (Figures 4G,H). Taken together, CFI was significantly upregulated in glioma tissues and cell lines and correlated with worse prognosis in GBM patients, indicating its potential as a prognostic biomarker.

### CFI Promotes Glioma Cell Proliferation, Invasion, and Migration *in vitro*

**Figure 5:**
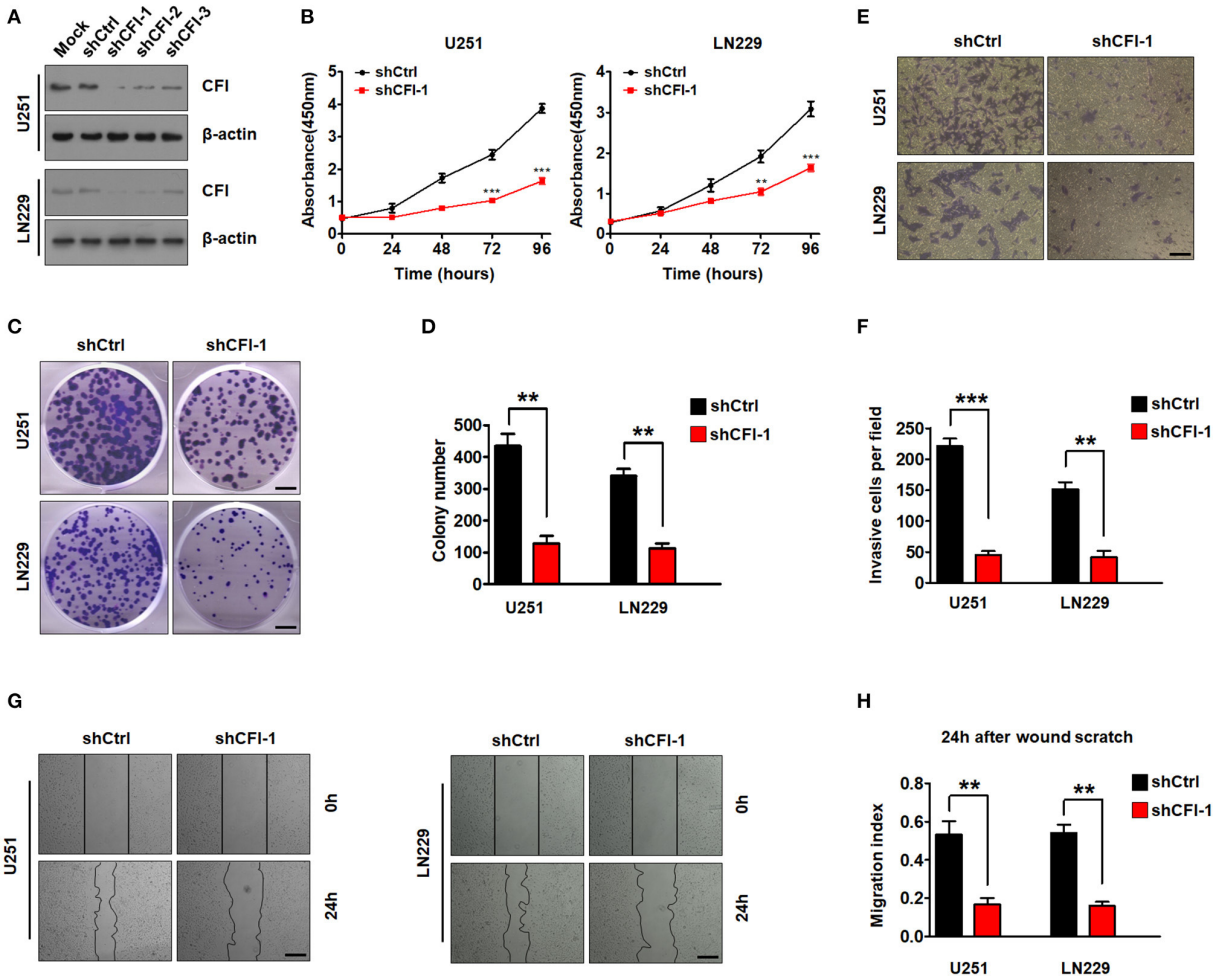
CFI in glioma suppresses tumor cells proliferation, invasion, and migration. **(A)** Immunoblot showing CFI protein levels in U251 and LN229 cells transduced with three independent CFI shRNA or control shRNA. β-actin served as the loading control. **(B)** Percentage of viable U251 and LN229 cells transduced with shCtrl or shCFI-1. **P < 0.01, ***P < 0.001. **(C,D)** Number of colonies formed by U251 and LN229 cells transfected with shCtrl or shCFI-1. All experiments were repeated three times. **P < 0.01. **(E,F)** Invasion capacity of U251 and LN229 cells transfected with shCFI-1 or shCtrl in the transwell assay is shown. **P < 0.01, ***P < 0.001, *n* = 3. **(G,H)** Wound coverage after 24 h by U251 and LN229 cells transfected with shCFI-1 or shCtrl. Quantification of wound healing assays is shown. **P < 0.01, *n* = 3.

To gain further insights into the role of CFI in gliomas, we knocked down its expression in U251 and LN229 glioma cell lines using three independent lentiviral shRNAs. As shown in Figure 5A, the cells transfected with shCFI constructs showed a significant reduction in CFI protein level compared to the control shRNA group. Furthermore, shCFI-1 showed the highest silencing efficiency (Figure 5A) and was used for the subsequent experiments. To determine whether inhibition of CFI can affect glioma development, we analyzed the proliferation, migration, and invasion capacities of the CFI-knockdown cells *in vitro*. The growth rates of CFI-depleted U251 and LN229 cells were significantly lower compared to the control cells (Figure 5B). Consistent with this, knocking down CFI also markedly reduced the number of colonies formed by the glioma cells (Figures 5C,D). Accumulating evidences showed that the invasion and migration of tumor cells to adjacent and distant tissues are the major hindrances in the treatment of glioma patients (Sanai and Berger, 2018). Therefore, we next evaluated the effect of CFI knockdown on the invasion and migration of glioma cells through transwell and wound healing assays, respectively. As shown in Figures 5E,F, CFI depletion significantly decreased the number of invading cells compared to the controls. Likewise, the wound coverage of the shCFI-transfected glioma cells was also significantly lower 24 h after plating, which was indicative of decreased migration potential compared to the control cells (Figures 5G,H). Collectively, these results suggest that downregulation of CFI effectively inhibits cell proliferation, invasion, and migration of glioma cells.

**Figure 6:**
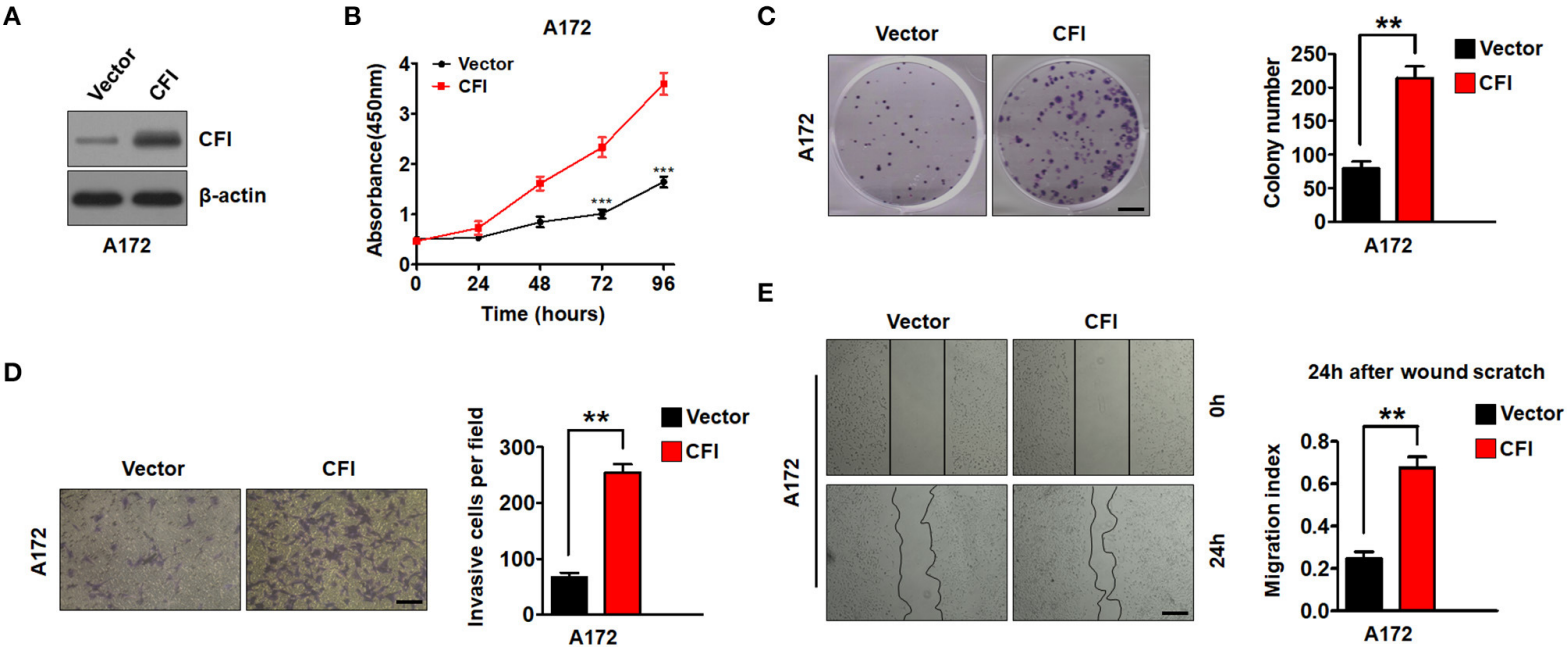
CFI overexpression enhances proliferation, invasion, and migration of glioma cells. **(A)** Immunoblot showing CFI protein levels in A172 cells transduced with CFI or vector control. β-actin served as the loading control. **(B)** Percentage of viable A172 cells transduced with CFI or vector control. ***P < 0.001. **(C)** Number of colonies formed by A172 cells transfected with CFI or vector control. All experiments were repeated three times independently. **P < 0.01. **(D)** Invasion capacity of A172 cells transfected with CFI or vector control in the transwell assay. **P < 0.01, *n* = 3. **(E)** Wound coverage after 24 h by A172 cells transfected with CFI or vector control. Quantification of wound healing assays is shown. **P < 0.01, *n* = 3.

To further establish an oncogenic role of CFI in glioma, we overexpressed the CFI gene in A172 cells (Figure 6A) and performed the same functional assays. As shown in Figure 6B, the A172 cells overexpressing CFI displayed a marked increase in growth rate compared to the control cells, as well as a significant increase in the number of colonies (Figure 6C). Consistent with these properties, ectopic expression of CFI in A172 cells also enhanced their invasiveness (Figure 6D) and the migration index in the wound healing assay (Figure 6E) compared to the control cells. Together, CFI overexpression induced proliferation, invasion, and migration in glioma cells, indicating that the gain of function of CFI likely enhances their malignant potential.

### CFI Knockdown Inhibits the Growth of Intracranial Glioma Xenografts

**Figure 7:**
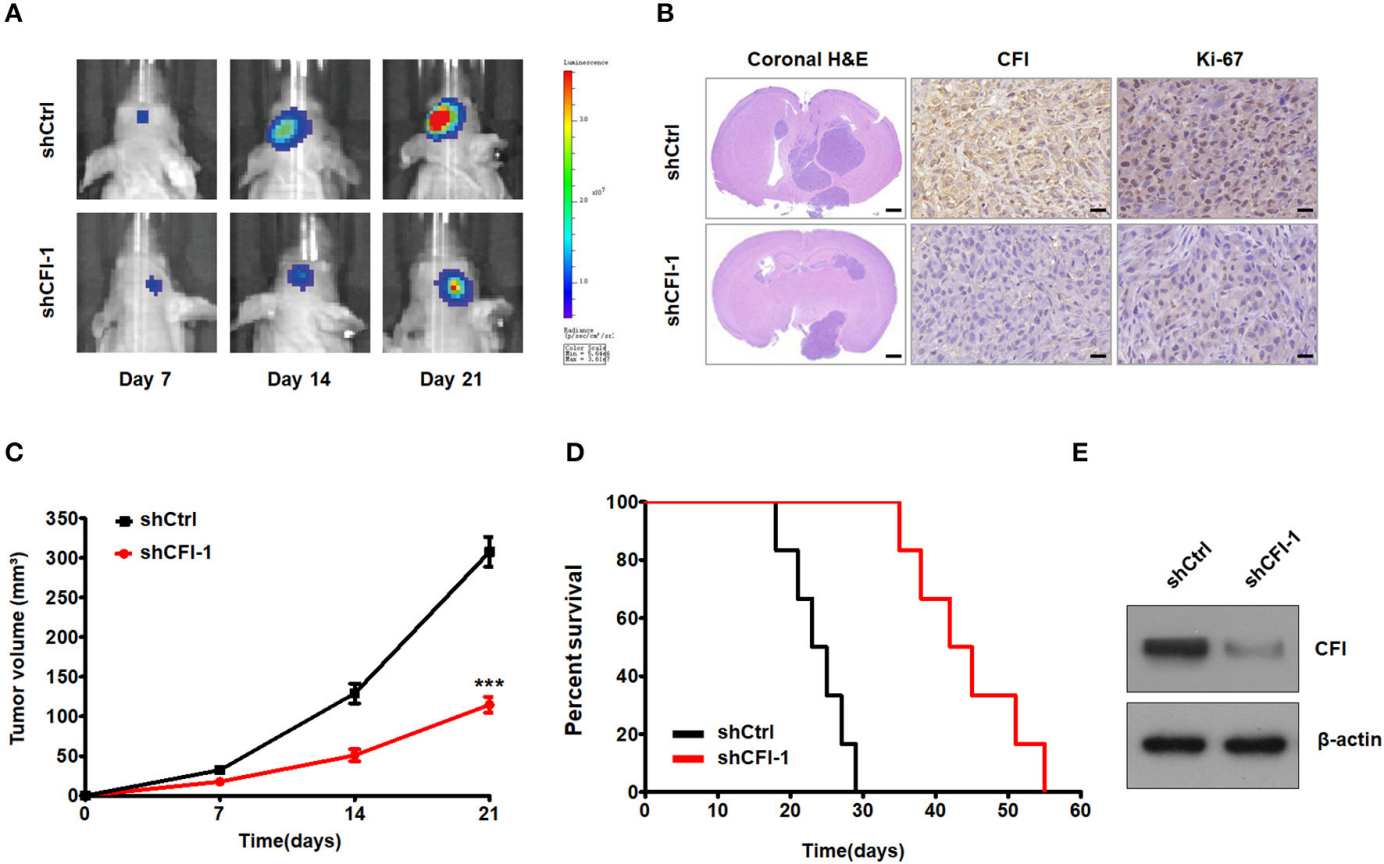
CFI depletion inhibits tumor growth *in vivo*. **(A)** Representative pseudocolor bioluminescence images of mice bearing control or shCFI U251 cell-derived intracranial xenograft on the indicated days. **(B)** Representative HandE staining for intracranial xenografts and IHC analysis to detect CFI and Ki-67 expression in intracranial xenografts in mice. Scale bar = 1 mm (first panels) and 400 μm (second and third panels). **(C)** Growth curves of control or shCFI U251 cell-derived intracranial xenografts (*n* = 6). ***P < 0.001. **(D)** Survival curves of the tumor-bearing mice (*n* = 6). **(E)** Immunoblot showing CFI expression level in control and shCFI xenografts. β-actin was used as the negative control.

To further explore the role of CFI in glioma progression *in vivo*, we injected luciferase-labeled control or shCFI U251 cells intracranially into 4-−6-week-old nude mice. Tumor growth was monitored using *in vivo* bioluminescence imaging system. The control mice exhibited tumor growth over 21 days after inoculation, whereas the CFI-depleted glioma cells formed significantly smaller tumors (Figure 7A). Representative H&E stainings for intracranial xenografts are shown in Figure 7B. Immunohistochemical staining showed decreased expression of CFI and Ki67 in CFI-knockdown tumors compared to control tumors in mice (Figure 7B). Quantification of tumor volume also indicated a marked deceleration in xenograft growth in the CFI-knockdown vs. control groups (Figure 7C). Consistent with these findings, Kaplan–Meier analysis demonstrated that the mice injected with shCFI-U251 cells survived considerably longer than those injected with the control U251 cells (Figure 7D). Finally, CFI protein levels were significantly lower in the xenografts derived from shCFI-U251 cells compared to control cells (Figure 7E). Together, inhibition of CFI impedes glioma growth *in vivo*, indicating its therapeutic potential.

## Discussion

Gliomas are the most prevalent primary malignancy of the brain in adults and are difficult to treat, resulting in a dismal 35% 5-year OS rate (Lapointe et al., 2018; Zachariah et al., 2018). The clinical outcome of the patients can improve with the development of accurate prognostic markers and therapeutic targets that allow personalized treatment. Several clinically significant molecular alterations (including mutations in IDH, 1p/19q, MGMT, and EGFR) have been described in gliomas and combined with histology for tumor classification (Eckel-Passow et al., 2015; Hoshide and Jandial, 2016; Louis et al., 2016; Jiang et al., 2020). In addition, novel molecular biomarkers have also been identified that can accurately predict prognosis and monitor therapeutic response in patients with glioma. Tachon et al. reported that the transcription factor Mesenchyme Homeobox 2 (MEOX2), a prognostic biomarker of LGG, is closely correlated to the overall survival (Tachon et al., 2019). Jiang et al. further demonstrated that low expression of ATP binding cassette subfamily C member 8 (ABCC8) is an independent predictor of favorable prognosis in glioma patients (Zhou et al., 2020). In this study, we found that CFI is significantly upregulated in glioma tissues and strongly correlated with the prognosis of glioma patients in TCGA and CGGA cohorts. Bioinformatics analyses also indicated a significant correlation of CFI expression level with several clinicopathological features and identified CFI as an independent prognostic indicator of OS in gliomas. CFI was also significantly upregulated in glioma cell lines and tumor tissues, and its ectopic expression enhanced the invasion, migration, and proliferation of tumor cells *in vitro*. Thus, CFI is a promising novel biomarker for stratifying glioma patients into prognostic groups.

At present, glioma is diagnosed on the basis of clinicopathological features, which also serve as the indicators of disease progression (Huse and Aldape, 2014; Wesseling et al., 2015; van den Bent et al., 2017). The WHO grade, IDH mutation, and 1p19q codeletion can effectively predict the prognosis of glioma patients. Several studies have shown that the combination of molecular biomarkers can supplement clinical features to improve the latter's predictive accuracy for cancer prognosis. In the current study, we found that higher WHO grade, wildtype IDH, 1p19q non-codeletion, higher histology grade, and older age correlated significantly with higher CFI expression, indicating that CFI is related to poor prognosis. Univariate and multivariate Cox regression analysis demonstrated that CFI is an independent prognostic indicator of OS in patients with gliomas. The respective AUC of the ROCs of different clinical variables indicated superior prognostic performance of CFI compared to IDH mutation status, 1p19q co-deletion status, or histology grade. A nomogram model integrating CFI and six clinicopathological variables showed moderate sensitivity and specificity in predicting the overall survival of glioma patients. Therefore, CFI is a highly promising biomarker for predicting glioma patient prognosis along with other indicators.

Functional annotation of CFI further revealed that the JAK/STAT, NOD-like receptor, pathways in cancer, T cell receptor, and VEGF pathways were significantly enriched in the CFI^high^ phenotype, indicating that CFI may drive glioma progression through the aforementioned signaling pathways. Furthermore, we provided experimental evidence that CFI may in part contribute to FAK activation in VEGF pathway. In addition, the ANXA1, ANXA2, C1R, C1S, CASP4, CP, PLAU, PROS1, SERPINA3, and TNFRSF12A genes were positively associated with CFI expression and are possibly involved in glioma progression as well. However, we did not identify the genes that are negatively correlated with CFI expression due to the limitation of gene correlation analysis. Nevertheless, silencing CFI in the glioma cells significantly decreased their proliferation, invasion, and migration abilities, whereas its ectopic expression had the opposite effect. Thus, CFI likely functions as an oncogene in glioma and promotes tumor progression. According to their clinicopathological and histological features, gliomas are classified into the IDH-mutant grade II glioma, IDH-wildtype grade II glioma, IDH-mutant and 1p/19q-codeleted grade III glioma, IDH-mutant and 1p/19q non-co-deleted grade III glioma, IDH-wildtype GBM, IDH-mutant GBM, and other subtypes (Chai et al., 2019b; Chang et al., 2020; Jiang et al., 2020). We also evaluated CFI expression and prognostic value in all aforementioned glioma subtypes in this study. Statistically significant survival differences were observed between CFI^high^ and CFI^low^ groups in IDH-mutant grade II glioma and IDH-mutant GBM in the CGGA dataset.

To summarize, integrated bioinformatics analysis showed that CFI is upregulated in gliomas and its expression level is correlated to the WHO tumor grade, IDH mutation status, and other clinical variables. In addition, we identified CFI as an independent prognostic factor of OS in glioma patients. Functional *in vitro* and *in vivo* assays further indicated that CFI knockdown suppressed glioma cell proliferation, invasion, and migration, whereas its overexpression had the opposite effects. Our findings provide novel molecular insights into glioma progression and recognize CFI as a promising prognostic biomarker and therapeutic target for glioma.

## Data Availability Statement

The raw data supporting the conclusions of this article will be made available by the authors, without undue reservation.

## Ethics Statement

The studies involving human participants were reviewed and approved by the ethics committee of Nanjing Medical University and Anhui Medical University. The patients/participants provided their written informed consent to participate in this study. The animal study was reviewed and approved by the Animal Welfare Ethical Review Committee of Nanjing Medical University and Anhui Medical University.

## Author Contributions

XC, YiW, and YuW conceived the project and designed the study. XC, WQ, MQ, SF, and CP performed the experiments. CP and JZ collected glioma and normal brain tissue samples. XC, WQ, MQ, and SF analyzed and/or interpreted the data. XC drafted the manuscript. YiW and YuW provided study supervision. All authors have read and approved the final manuscript.

## Conflict of Interest

The authors declare that the research was conducted in the absence of any commercial or financial relationships that could be construed as a potential conflict of interest.
