# Supplementary material for: A Candidate Prognostic Biomarker Complement Factor I Promotes Malignant Progression in Glioma
Source: Front Cell Dev Biol. 2021 Feb 4;8:615970. doi: 10.3389/fcell.2020.615970 (PMC7889977; doi:10.3389/fcell.2020.615970)
Supplement: Supplementary file 1 [file Table_1.DOCX]

**Supplementary Table 1.** Gene sets enriched in CFI^high^ gliomas in CGGA dataset.

| **Gene set name** | **NES** | **NOM p-val** | **FDR q-val** |
| --- | --- | --- | --- |
| KEGG_JAK_STAT_SIGNALING_PATHWAY | 1.915 | 0.002 | 0.012 |
| KEGG_NOD_LIKE_RECEPTOR_SIGNALING_PATHWAY | 1.904 | 0.000 | 0.012 |
| KEGG_PATHWAYS_IN_CANCER | 1.783 | 0.000 | 0.030 |
| KEGG_T_CELL_RECEPTOR_SIGNALING_PATHWAY | 1.814 | 0.002 | 0.024 |
| KEGG_VEGF_SIGNALING_PATHWAY | 1.804 | 0.000 | 0.025 |

NES: normalized enrichment score; NOM: nominal; FDR: false discovery rate. A normalized p-value < 0.05 with a FDR q-value < 0.05 were considered statistically significant.
