# Supplementary material for: A Candidate Prognostic Biomarker Complement Factor I Promotes Malignant Progression in Glioma
Source: Front Cell Dev Biol. 2021 Feb 4;8:615970. doi: 10.3389/fcell.2020.615970 (PMC7889977; doi:10.3389/fcell.2020.615970)
Supplement: Supplementary file 2 [file Table_2.DOCX]

**Supplementary Table 2.** The clinical and pathological characteristics for 134 GBM patients.

Characteristics Value

| Total samples (n) | 134 |
| --- | --- |
| Sex (n) |  |
| Male | 70 |
| Female | 64 |
| Medium age, years (range) | 56(19-72) |
| Tumor location |  |
| Frontal | 49 |
| Non-frontal | 85 |
| Medium KPS (range) | 80 (30-90) |
| MGMT promotor status |  |
| Methylated | 60 |
| Unmethylated | 74 |
| IDH genotype |  |
| Mutation | 25 |
| Wild-type | 109 |
| EBRT dose (Gy) | 60 |
| TMZ dose | 75 mg/m^2^/d after first surgery |

KPS: Karnofsky performance status; MGMT: O-6-methylguanine-DNA-methyltransferase; IDH: isocitrate dehydrogenase; EBRT: external-beam radiotherapy; TMZ: temozolomide.
