# Supplementary figures and images for: A Candidate Prognostic Biomarker Complement Factor I Promotes Malignant Progression in Glioma
Source: Front Cell Dev Biol. 2021 Feb 4;8:615970. doi: 10.3389/fcell.2020.615970 (PMC7889977; doi:10.3389/fcell.2020.615970)

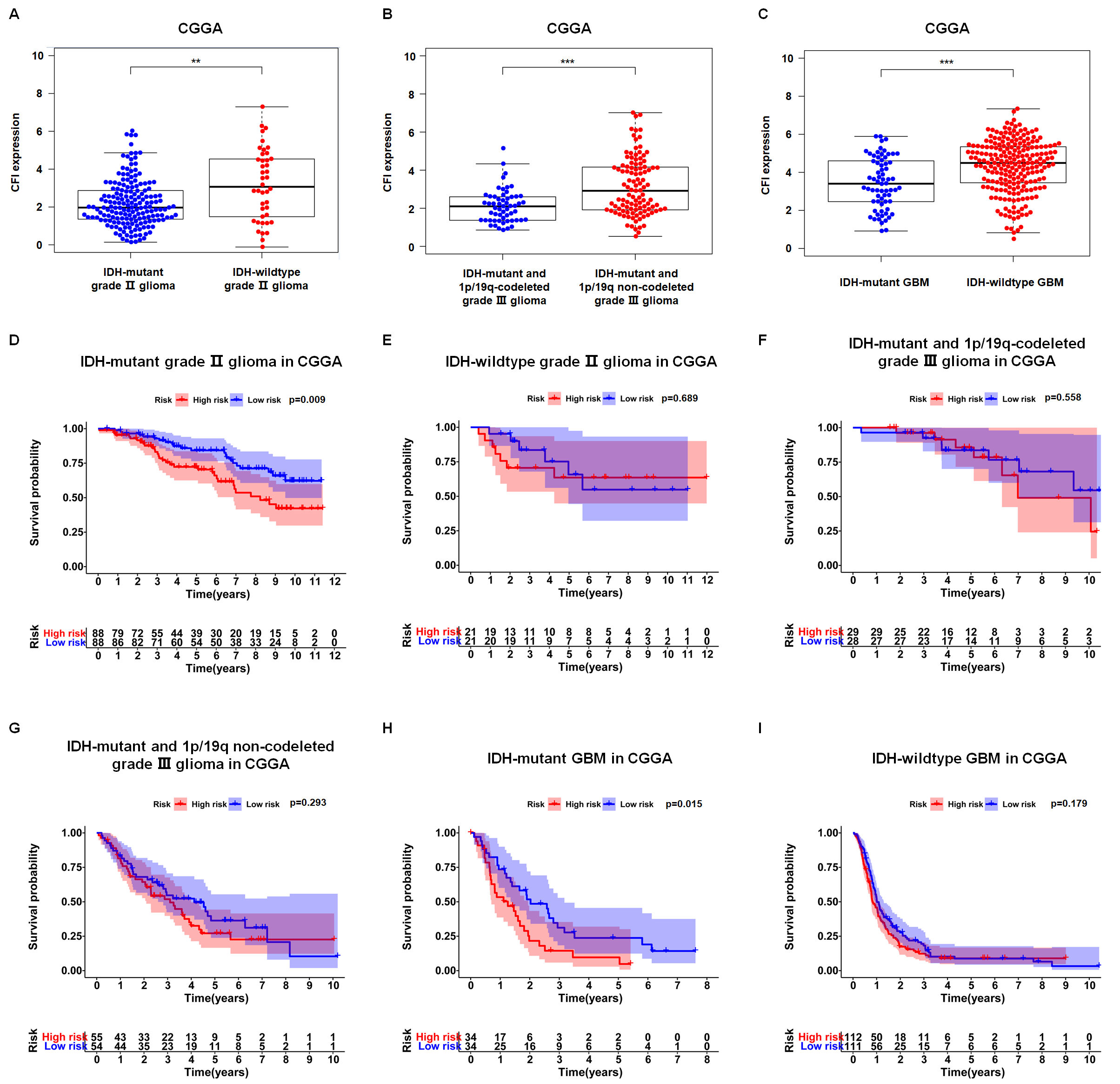

Supplement: Supplementary file 3 [file Image_1.TIF]

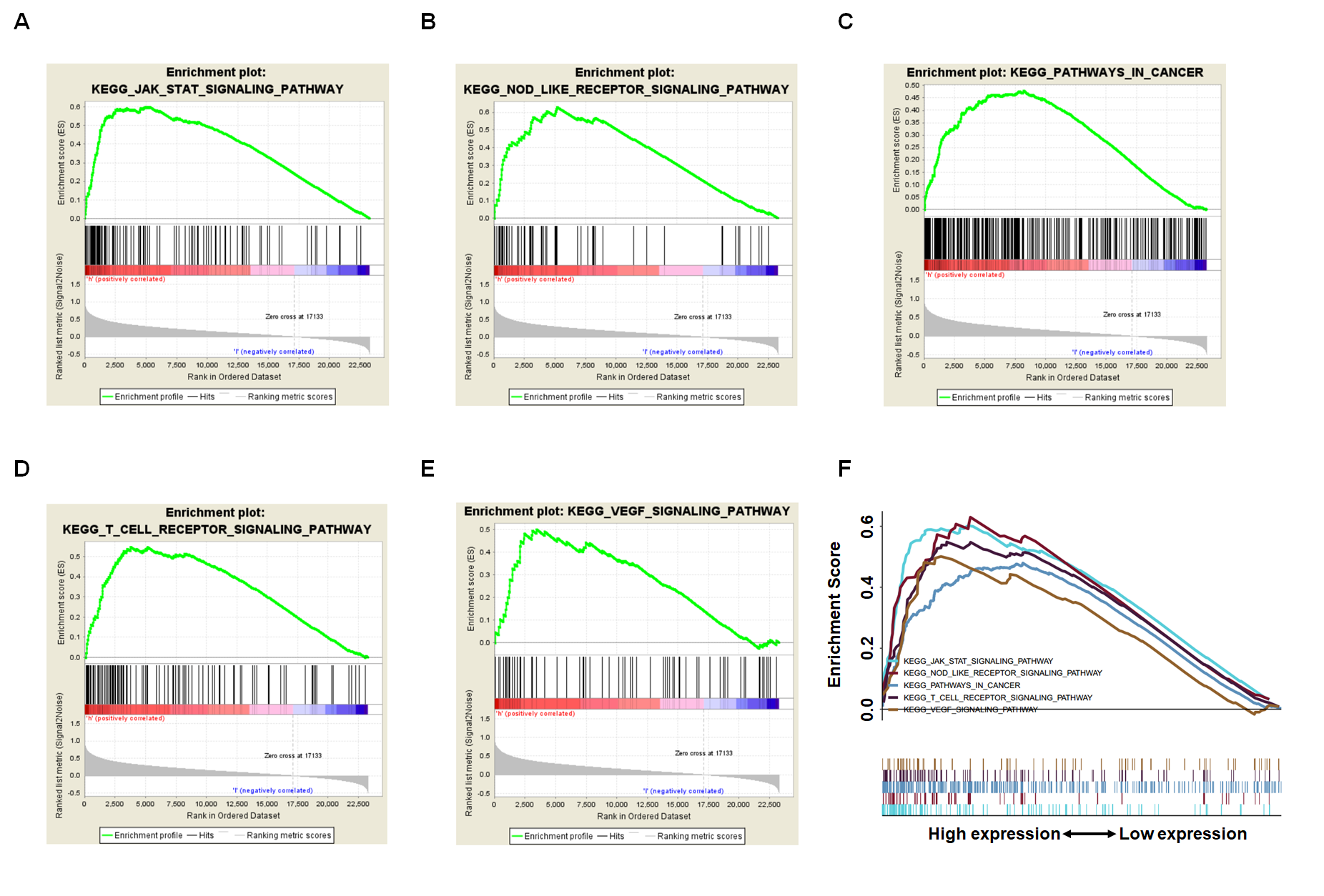

Supplement: Supplementary file 4 [file Image_2.TIF]

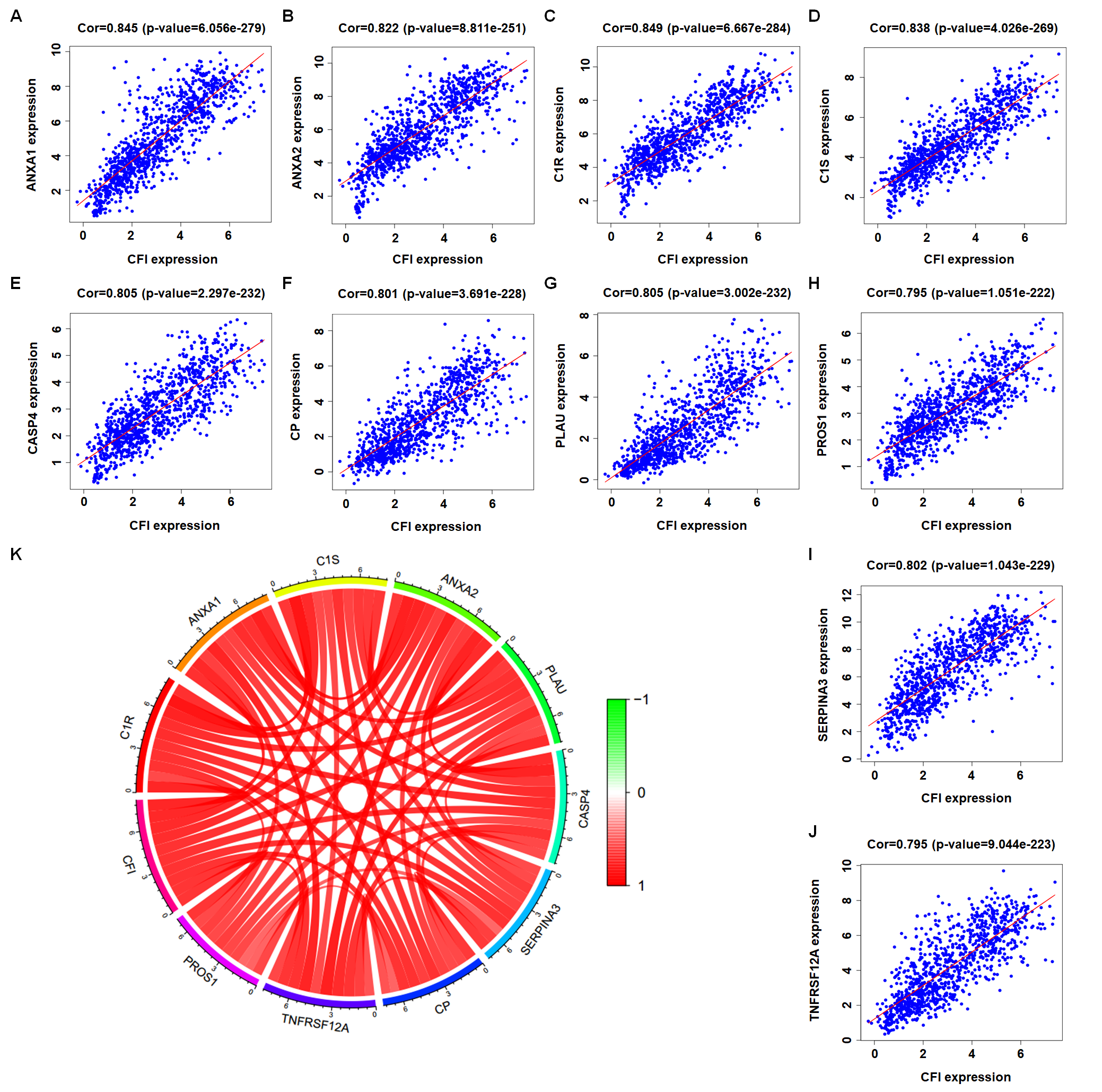

Supplement: Supplementary file 5 [file Image_3.TIF]

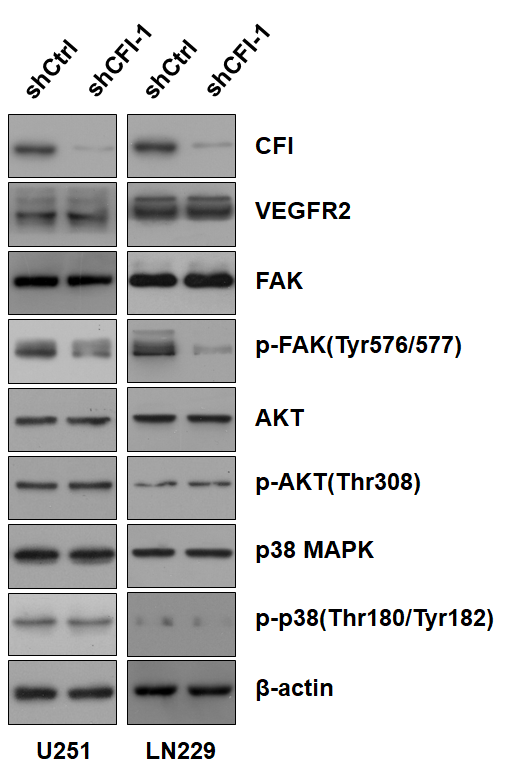

Supplement: Supplementary file 6 [file Image_4.TIF]
